# Supplementary material for: Regulating ion affinity and dehydration of metal-organic framework sub-nanochannels for high-precision ion separation
Source: Nat Commun. 2024 Mar 8;15:2145. doi: 10.1038/s41467-024-46378-6 (PMC10924084; doi:10.1038/s41467-024-46378-6)
Supplement: Supplementary file 1 — Supplementary information [file 41467_2024_46378_MOESM1_ESM.pdf]

Supplementary Information

Regulating ion affinity and dehydration of metal-organic framework sub-nanochannels for high-precision ion separation

Ri-Jian Mo[1], Shuang Chen[1], Li-Qiu Huang[1], Xin-Lei Ding[1], Saima Rafique[1], Xing-Hua Xia[1*] & Zhong-Qiu Li[1*]

[1]State Key Laboratory of Analytical Chemistry for Life Science, School of Chemistry and Chemical Engineering, Nanjing University, 210023 Nanjing, China

*E-mail: xhxia@nju.edu.cn; zhongqiuli@nju.edu.cn

1. Supplementary Figures

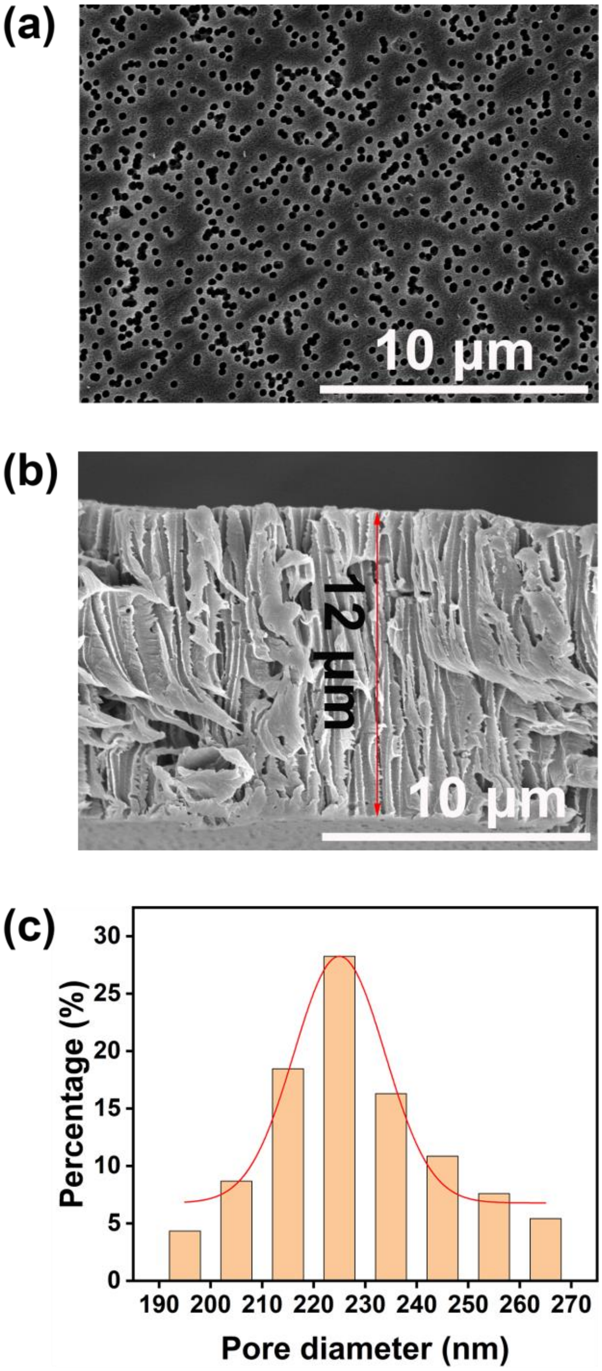

Supplementary Fig. 1 SEM images of a PET membrane. (a) Top view of the membrane. (b) Cross-sectional view of the membrane. (c) Pore diameter distribution of PET membrane with an average value of 227±16.7 nm.

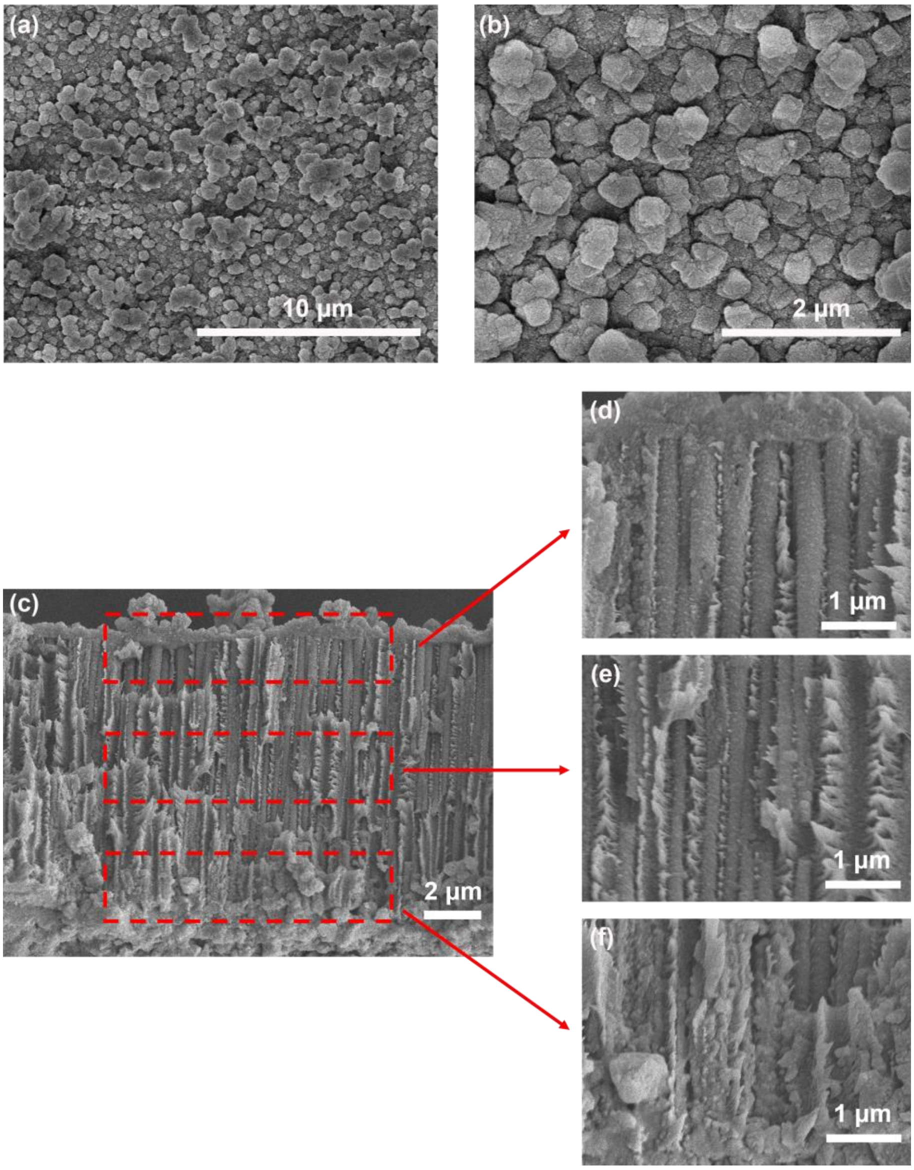

Supplementary Fig. 2 SEM images of a UiO-66 membrane. (a-b) Top view of the membrane. (c-f) Cross-sectional view of the membrane.

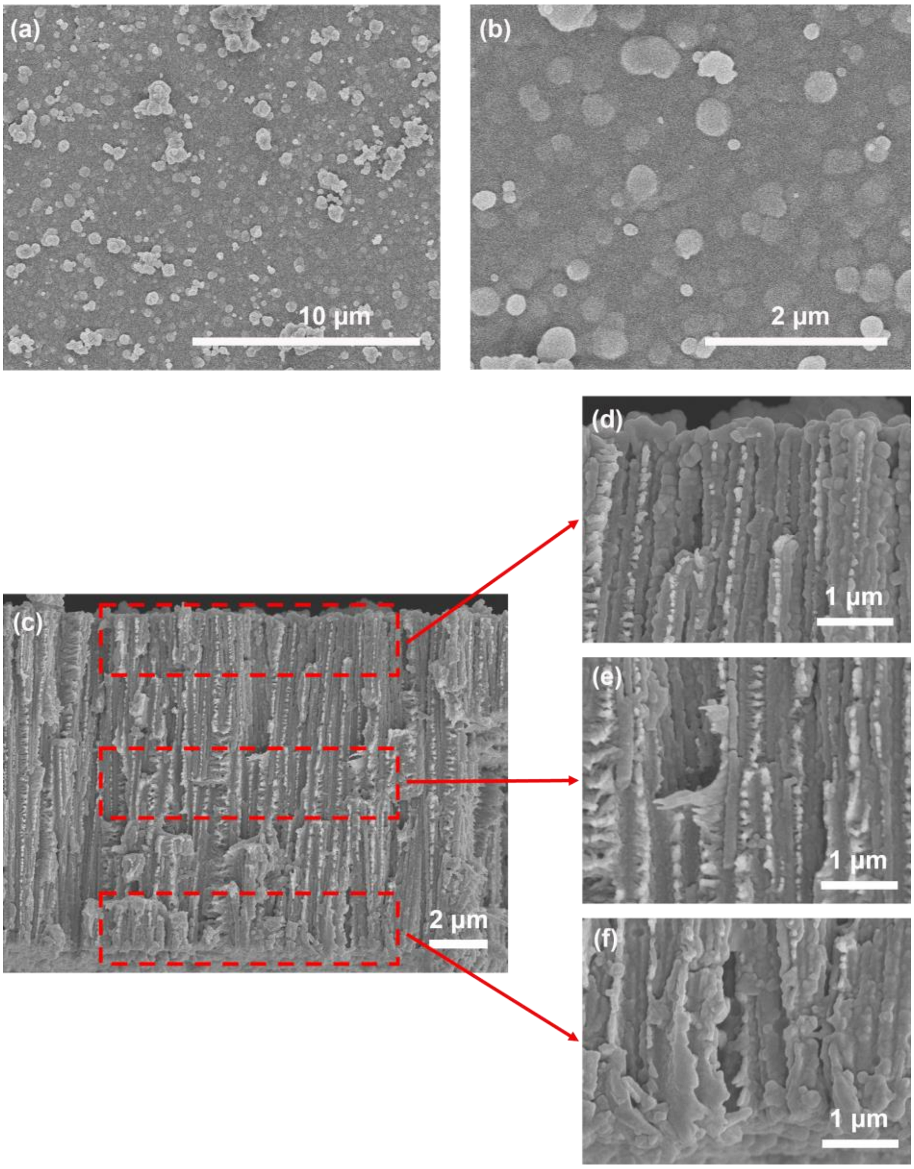

Supplementary Fig. 3 SEM images of a UiO-66-$(OH)_2$ membrane. (a-b) Top view of the membrane. (c-f) Cross-sectional view of the membrane.

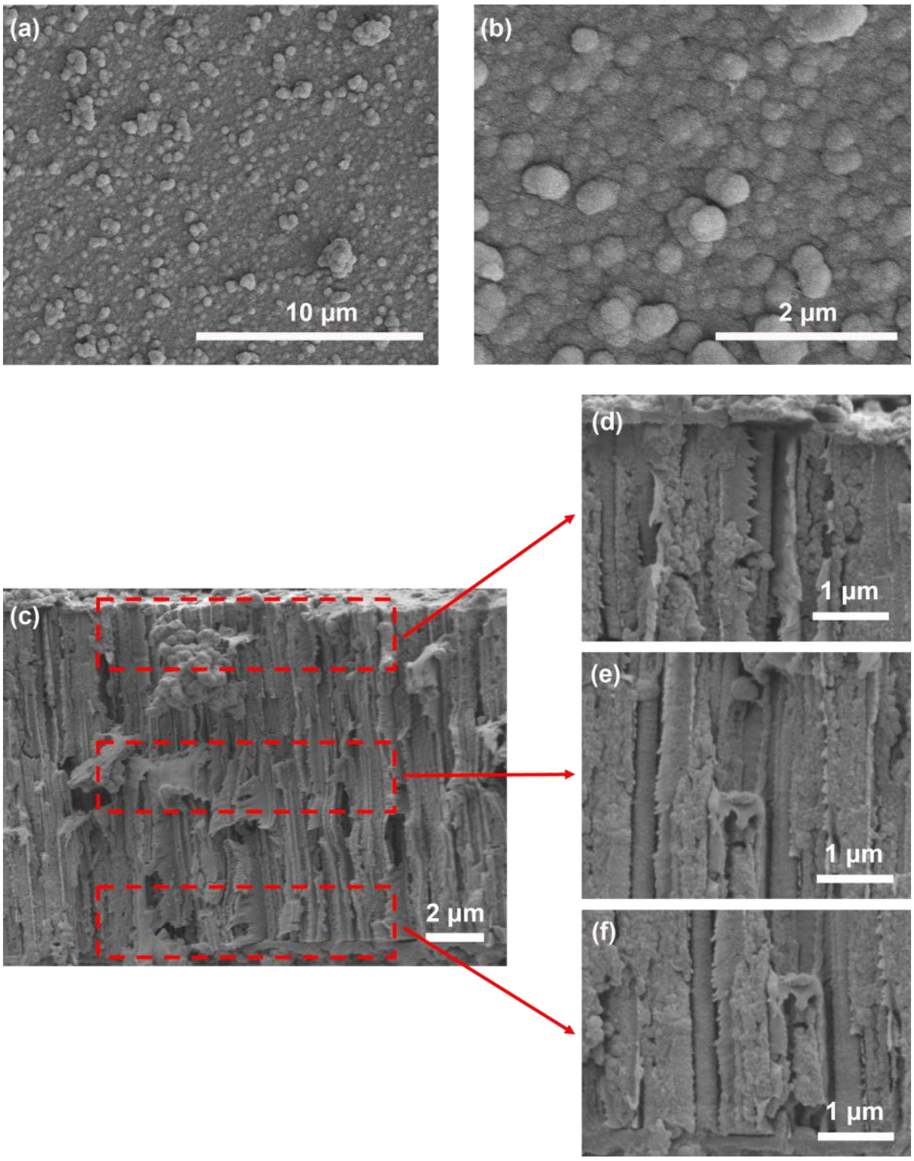

Supplementary Fig. 4 SEM images of a UiO-66-$(SH)_2$ membrane. (a-b) Top view of the membrane. (c-f) Cross-sectional view of the membrane.

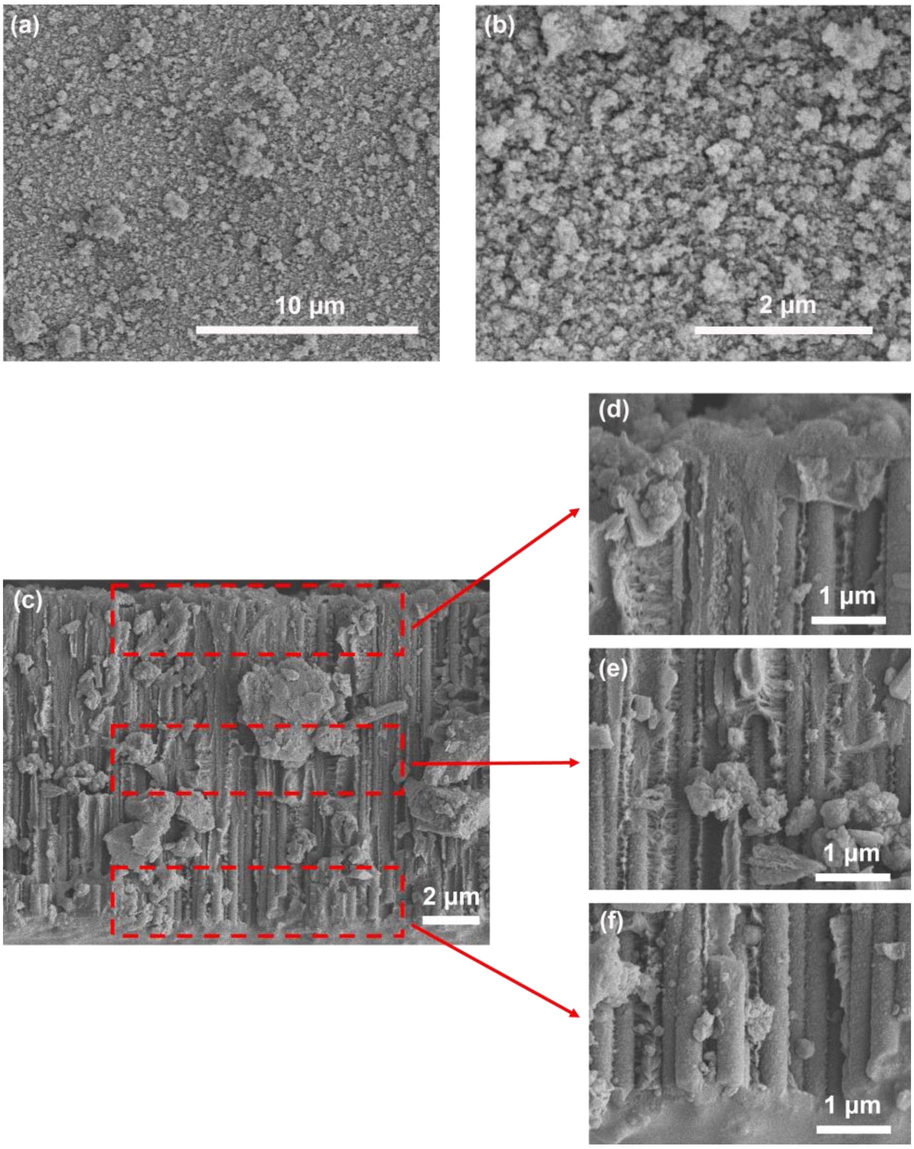

Supplementary Fig. 5 SEM images of a UiO-66-$(NH_2)_2$ membrane. (a-b) Top view of the membrane. (c-f) Cross-sectional view of the membrane.

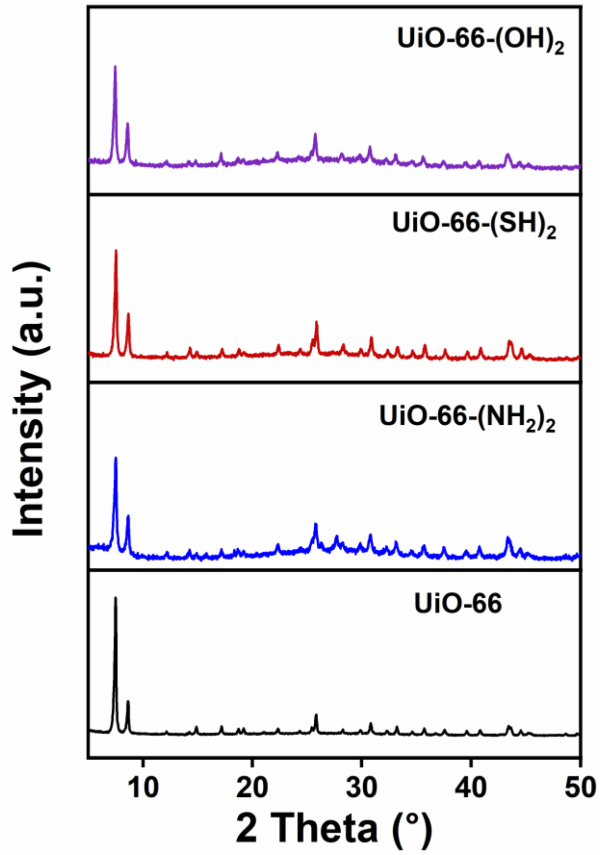

Supplementary Fig. 6 XRD patterns of UiO-66 and UiO-66-$(X)_2$ (X=OH, SH, NH_2) powders.

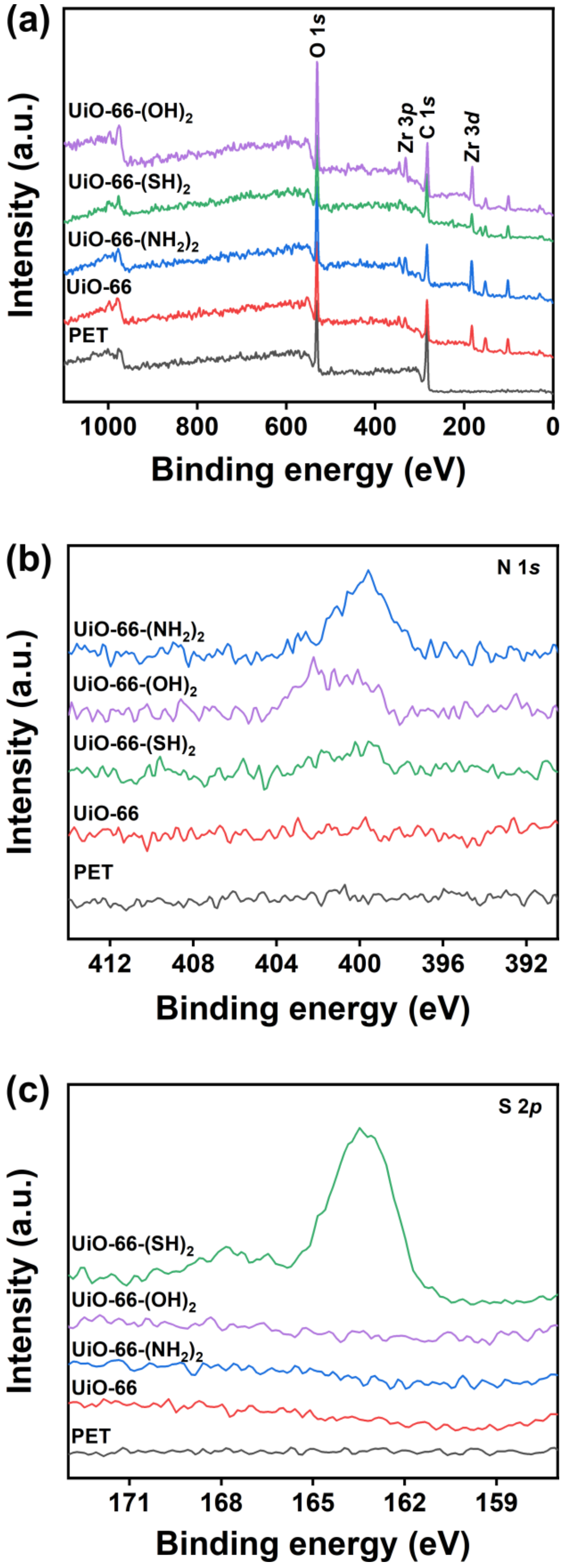

Supplementary Fig. 7 XPS spectra of the PET, UiO-66 and UiO-66-(X)$_2$ membranes. (a) full view; (b) N 1*s*; (c) S 2*p*.

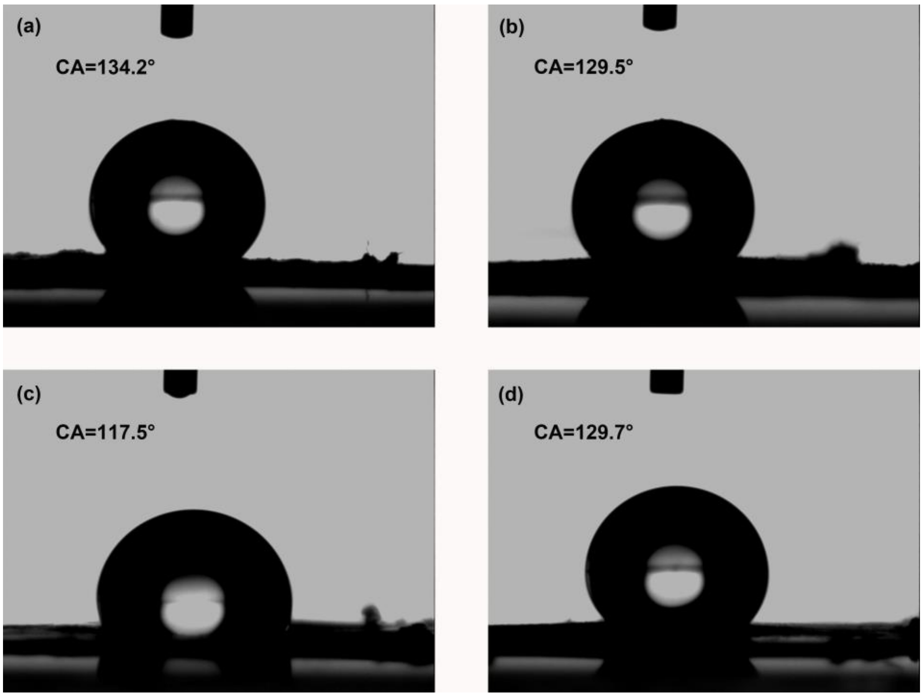

Supplementary Fig. 8 Contact angles of the UiO-66 and UiO-66-(X)$_2$ membranes.

(a) UiO-66; (b) UiO-66-(NH_2)$_2$; (c) UiO-66-(SH)$_2$; (d) UiO-66-(OH)$_2$.

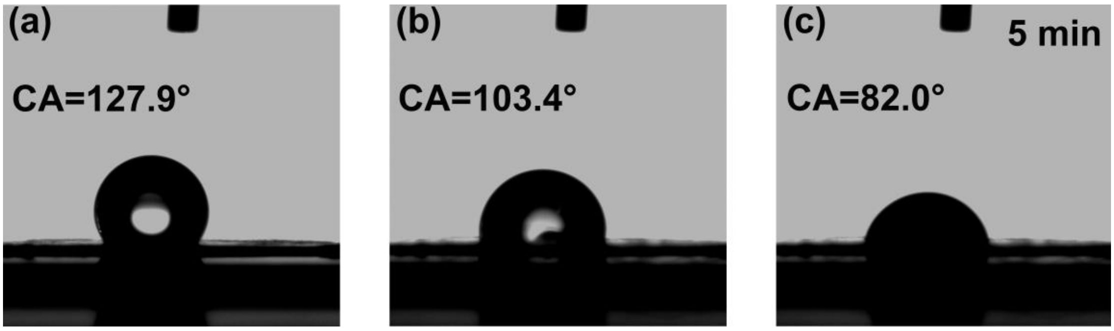

Supplementary Fig. 9 Contact angle of the UiO-66-$(OH)_2$ membrane before and after ethanol wetting. (a) Contact angle of the as-prepared membrane. (b) Contact angle of the membrane after ethanol wetting. (c) Contact angle of (b) after 5 min.

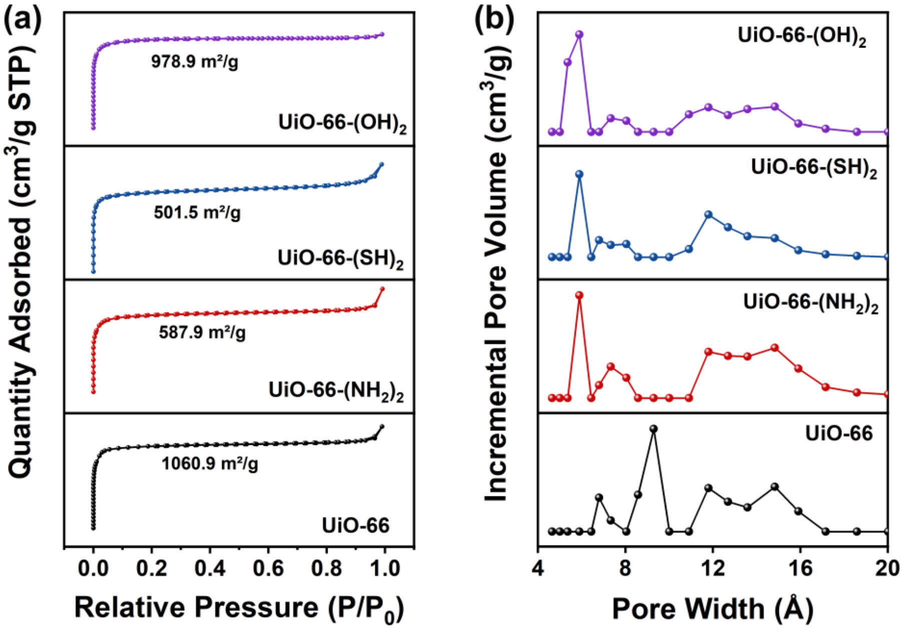

Supplementary Fig. 10 N_2 adsorption-desorption isotherm (a) and pore size (b) of UiO-66 and UiO-66-$(X)_2$ (X=OH, SH, NH_2).

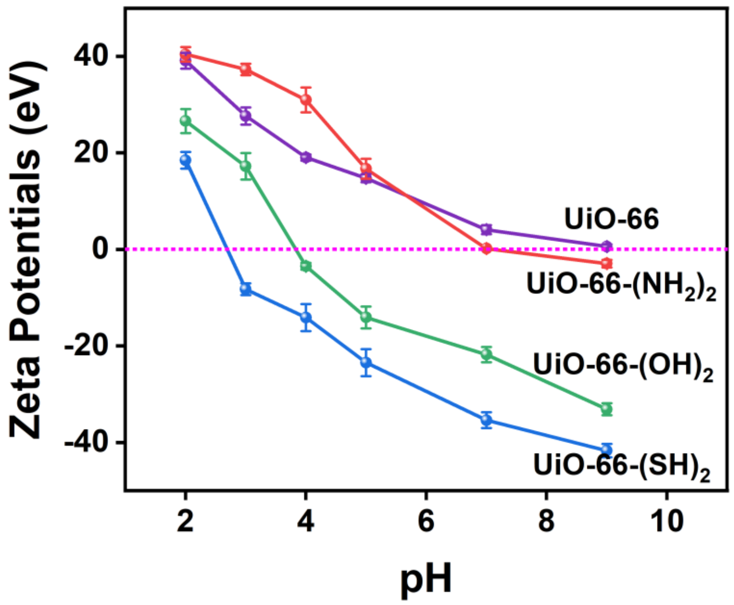

Supplementary Fig. 11 Zeta potentials of the UiO-66-$(X)_2$ (X= SH, NH_2 and OH) powders in solutions with different pH values (2.00, 3.00, 4.00, 5.00, 7.00 and 9.00). The error bars in this figure represent the standard deviation of three parallel tests.

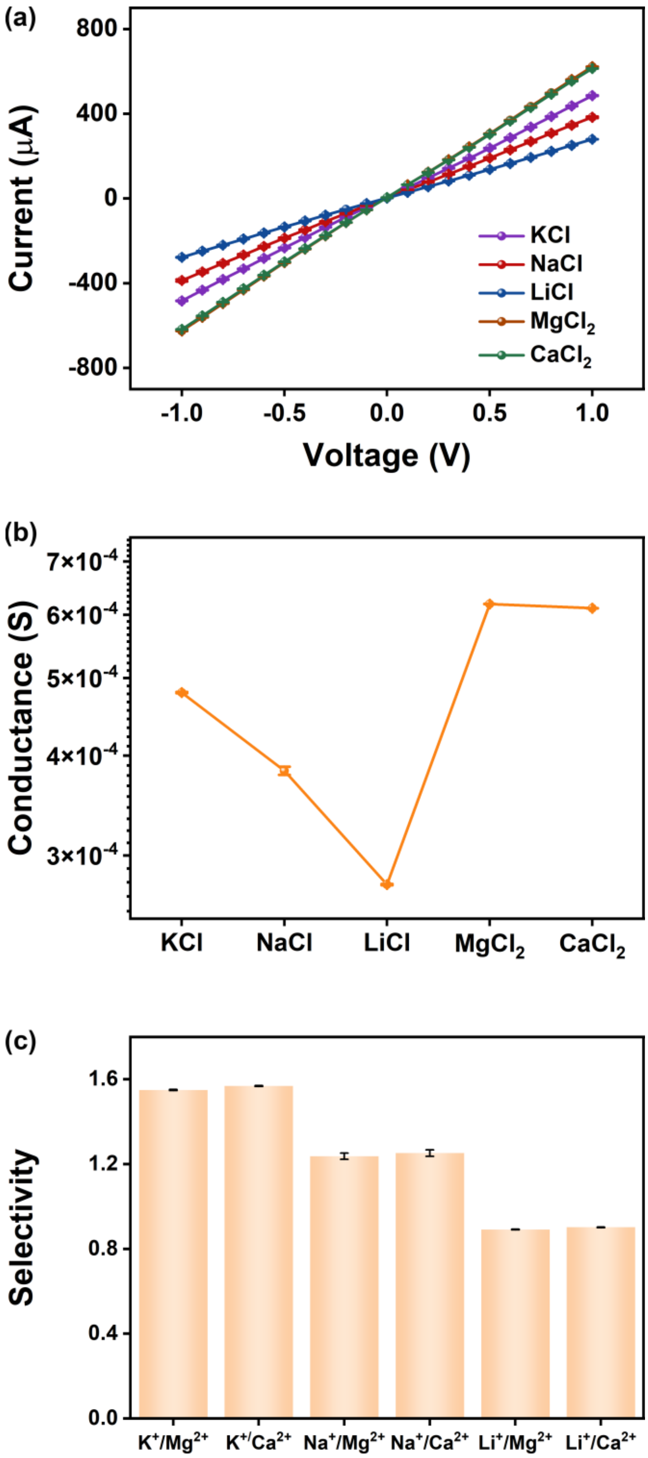

Supplementary Fig. 12 I-V curves (a), ionic conductance (b) of a PET membrane in different 100 mM electrolyte solutions. (c) Ion selectivity of the PET membrane. The error bars in all figures represent the standard deviation of three parallel tests.

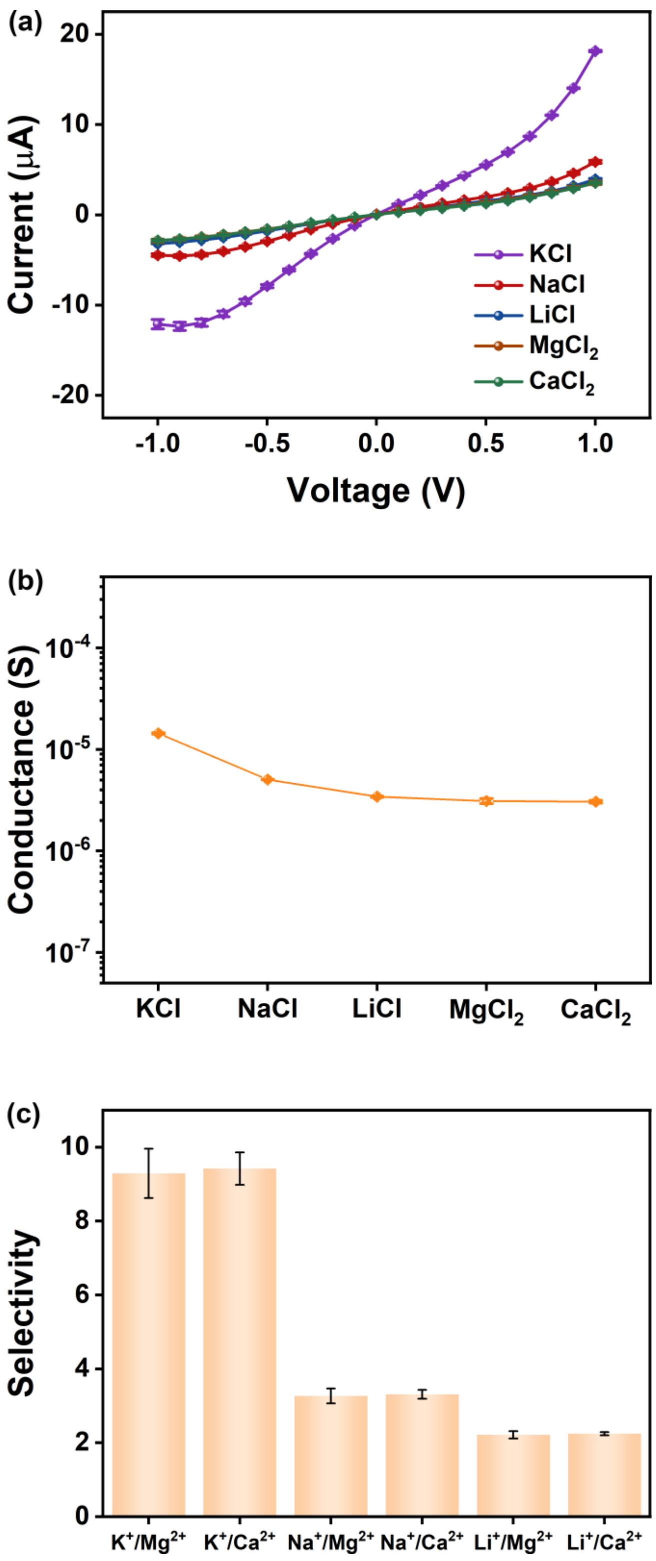

Supplementary Fig. 13 I-V curves (a), ionic conductance (b) of a UiO-66 membrane in different 100 mM electrolyte solutions. (c) Ion selectivity of the UiO-66 membrane. The error bars in all figures represent the standard deviation of three parallel tests.

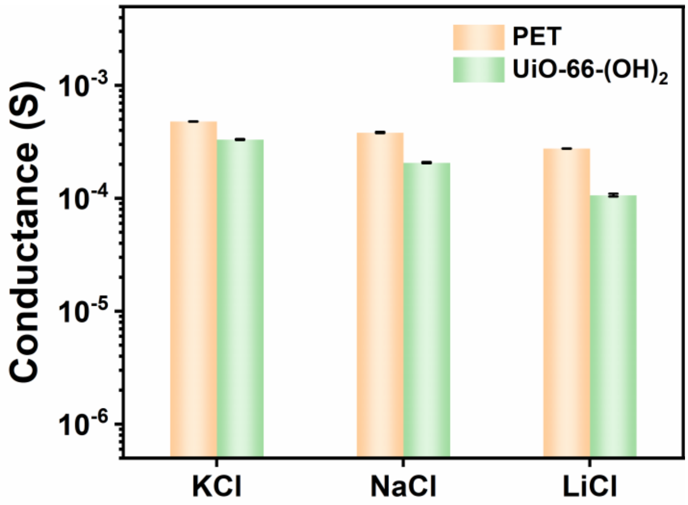

Supplementary Fig. 14 Conductance of monovalent ions in UiO-66-$(OH)_2$ and PET membrane. The error bars in this figure represent the standard deviation of three parallel tests.

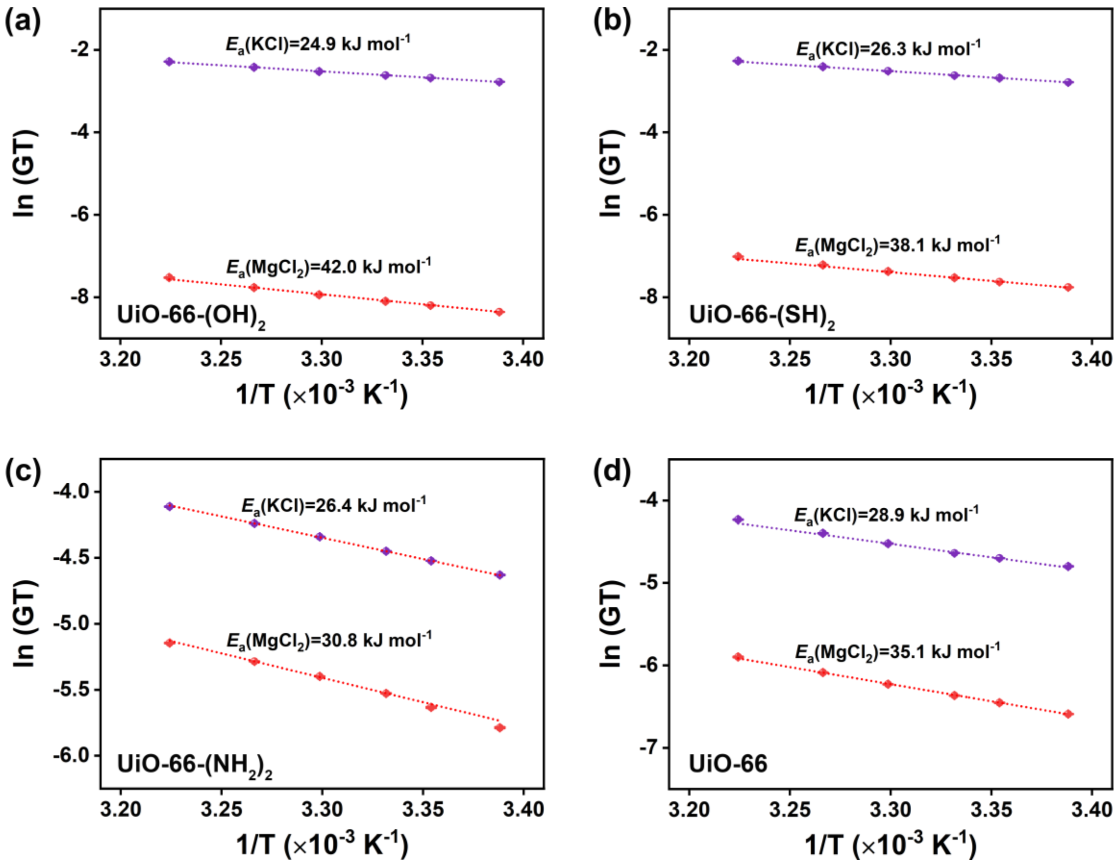

Supplementary Fig. 15 Arrhenius plots of the UiO-66-(X)$_2$ membranes in 100 mM KCl and MgCl$_2$ solutions. (a) UiO-66-(OH)$_2$ membrane; (b) UiO-66-(SH)$_2$ membrane; (c) UiO-66-(NH$_2$)$_2$ membrane; (d) UiO-66 membrane. The error bars in all figures represent the standard deviation of three parallel tests.

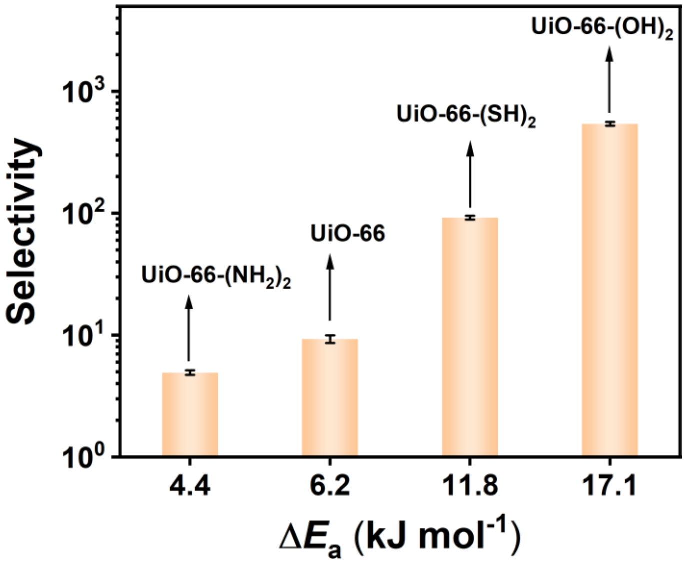

Supplementary Fig. 16 K^+/Mg^{2+} ion selectivity at different activation energy differences (ΔE_a) (that is, ΔE_a of UiO-66 and UiO-66-(X)$_2$ (X=NH_2, SH, OH) membranes) in 100 mM electrolyte solutions. Here, $\Delta E_a = E_a(MgCl_2) - E_a(KCl)$. The error bars in this figure represent the standard deviation of three parallel tests.

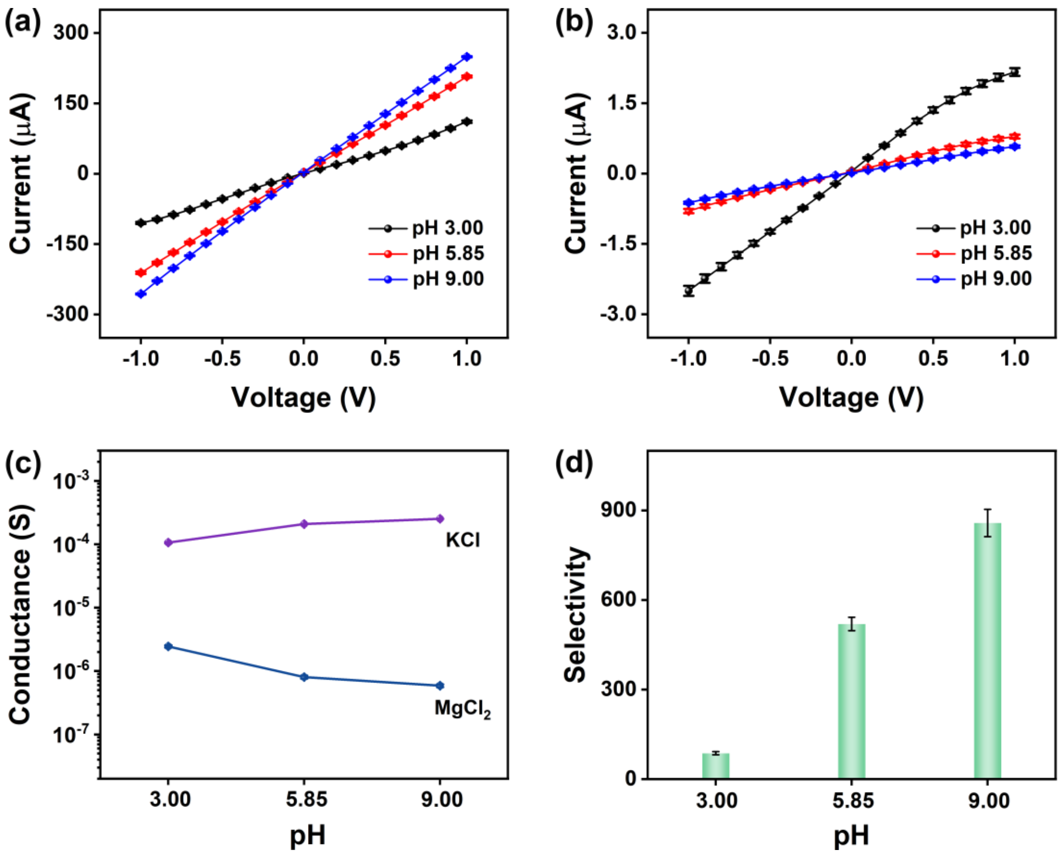

Supplementary Fig. 17 Influence of pH on the ion selectivity of a UiO-66-$(OH)_2$ membrane. I-V curves of the UiO-66-$(OH)_2$ membrane in 100 mM KCl (a) and $MgCl_2$ (b) solutions with different pH values. (c) Ionic conductance of the UiO-66-$(OH)_2$ membrane in 100 mM KCl and $MgCl_2$ solutions with different pH values. (d) K^+/Mg^{2+} selectivity of the UiO-66-$(OH)_2$ membrane under different pH values. The error bars in all figures represent the standard deviation of three parallel tests.

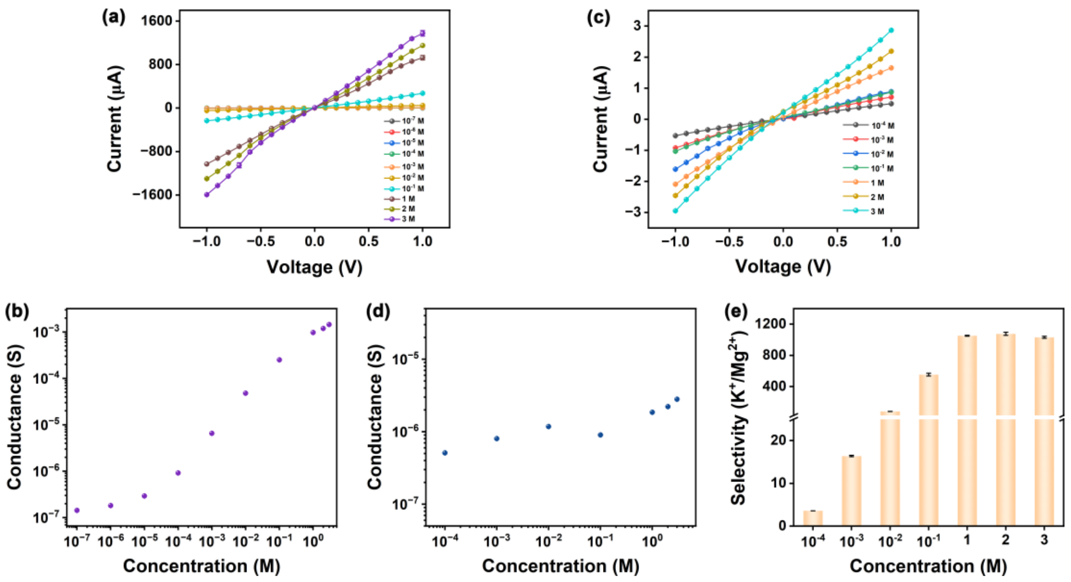

Supplementary Fig. 18 Relationship between ion selectivity and ion concentration in UiO-66-$(OH)_2$ membranes. (a) I-V curves of a UiO-66-$(OH)_2$ membrane in KCl solutions with various concentrations. (b) Ionic conductance of the UiO-66-$(OH)_2$ membrane in KCl with various concentrations (10^{-7}-3 M). (c) I-V curves of a UiO-66-$(OH)_2$ membrane in $MgCl_2$ solutions with various concentrations. (d) Ionic conductance of the UiO-66-$(OH)_2$ membrane in KCl with various concentrations (10^{-4}-3 M). (e) K^+/Mg^{2+} ion selectivity of UiO-66-$(OH)_2$ membrane at various concentrations (10^{-4}-3 M). The error bars in all figures represent the standard deviation of three parallel tests.

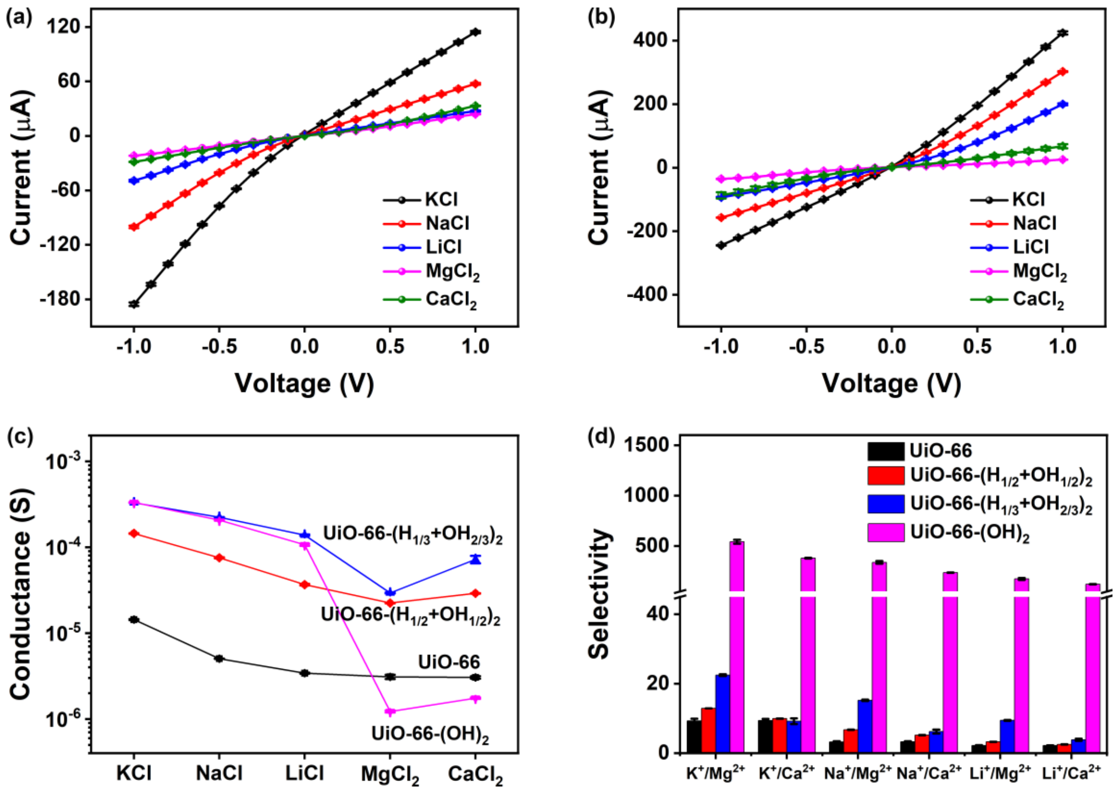

Supplementary Fig. 19 Influence of content of -OH group on ion selectivity of UiO-66-$(OH)_2$ membrane. (a) I-V Curves of a UiO-66-$(H_{1/2}+OH_{1/2})_2$ membrane. (b) I-V Curves of a UiO-66-$(H_{1/3}+OH_{2/3})_2$ membrane. (c) Ion conductance of the MOF membrane with different contents of -OH group. (d) Ion selectivity of the MOF membrane with different contents of -OH group. The error bars in all figures represent the standard deviation of three parallel tests.

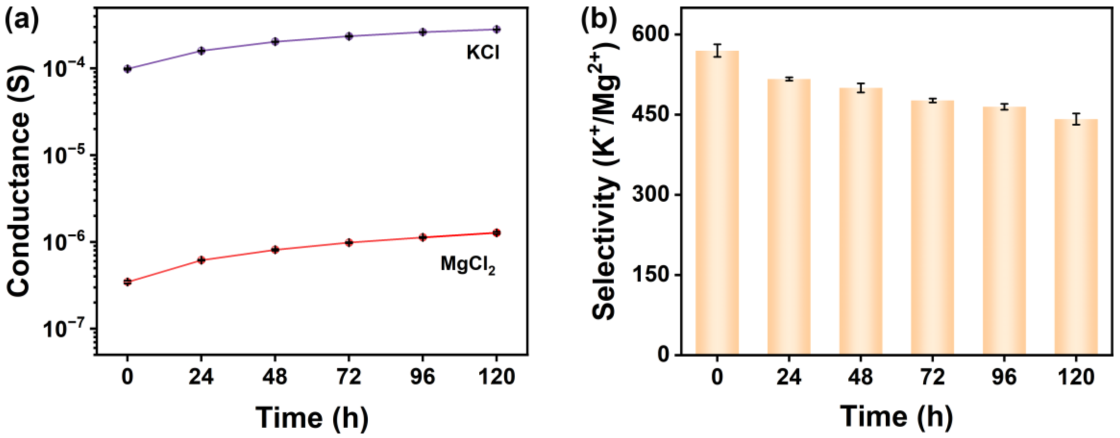

Supplementary Fig. 20 Stability of ion separation performance of UiO-66-$(OH)_2$ membrane. (a) Relationship between ion conductance and time. (b) Relationship between ion selectivity and time. The error bars in all figures represent the standard deviation of three parallel tests.

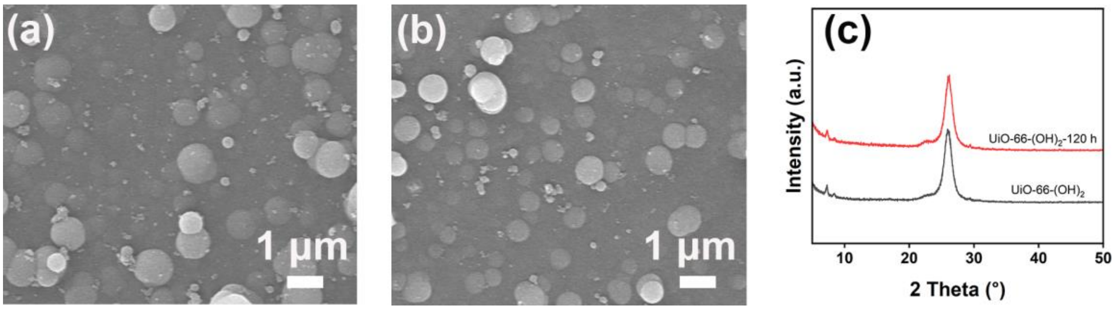

Supplementary Fig. 21 SEM images and XRD patterns of UiO-66-$(OH)_2$ membrane after the stability test. (a) Initial surface morphology of the membrane. (b) Surface morphology of the membrane after 120 h stability test. (c) XRD pattern of the membrane before and after stability test.

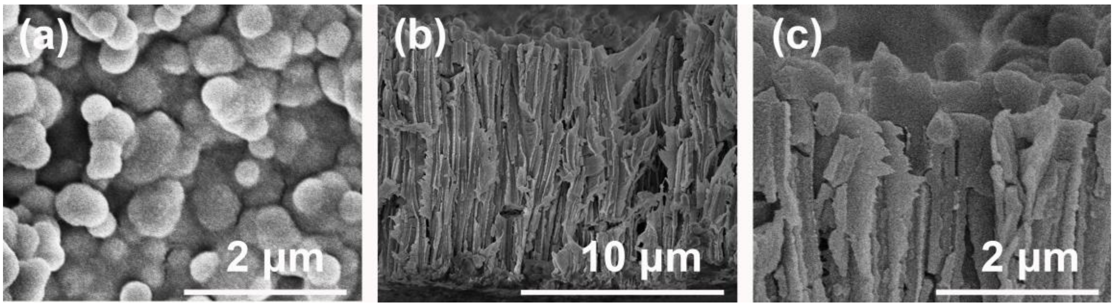

Supplementary Fig. 22 SEM images of a UiO-66-$(OMe)_2$ membrane. (a) Top view of the membrane. (b-c) Cross-sectional view of the membrane.

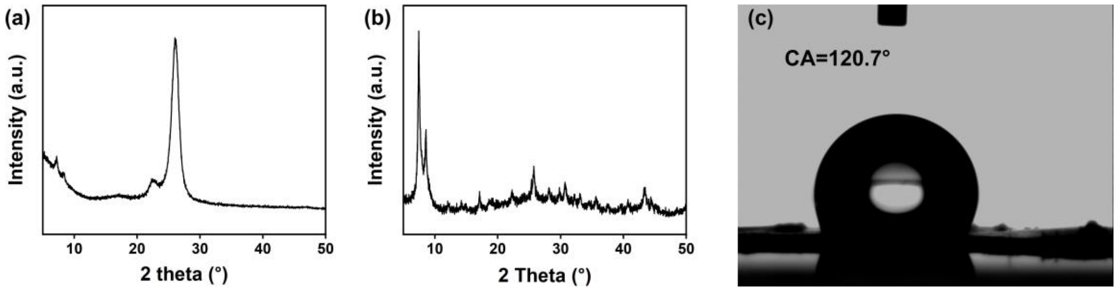

Supplementary Fig. 23 XRD patterns of (a) UiO-66-$(OMe)_2$ membrane and (b) UiO-66-$(OMe)_2$ powder. (c) Contact angle of a UiO-66-$(OMe)_2$ membrane.

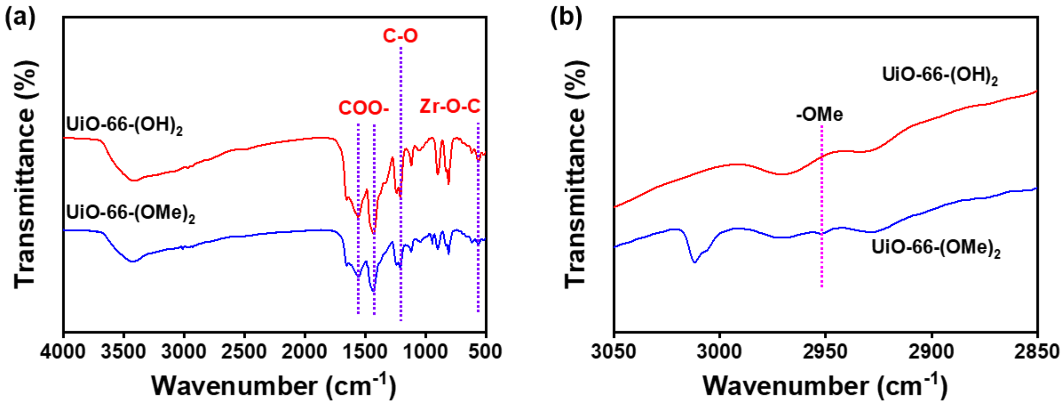

Supplementary Fig. 24 FTIR spectra of UiO-66-$(OH)_2$ and UiO-66-$(OMe)_2$. (a) full spectra; (b) enlarged spectra.

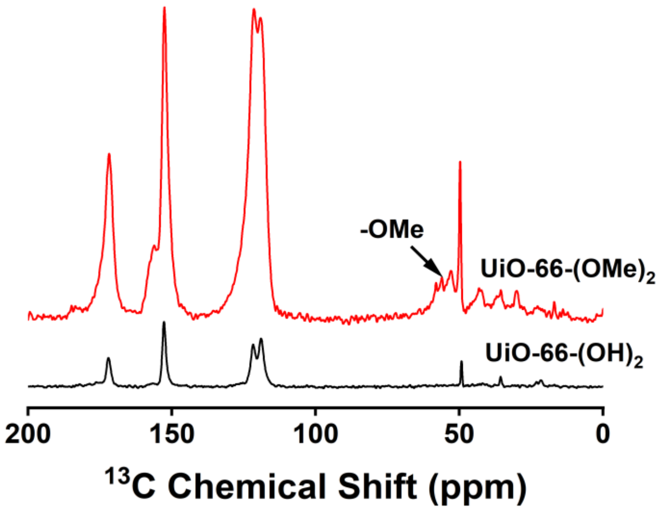

Supplementary Fig. 25 Solid state ^{13}C NMR spectra of UiO-66-$(OH)_2$ and UiO-66-$(OMe)_2$.

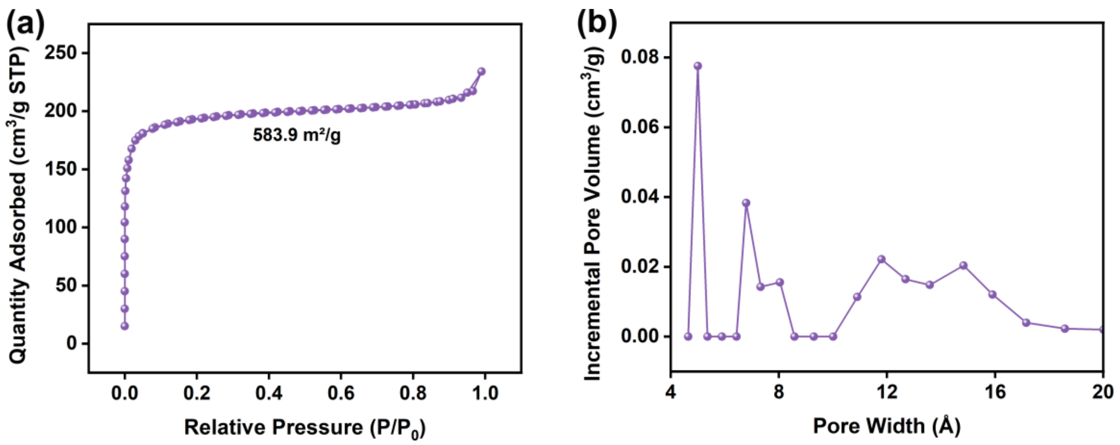

Supplementary Fig. 26 N_2 adsorption-desorption isotherm (a) and pore size (b) of UiO-66-$(OMe)_2$.

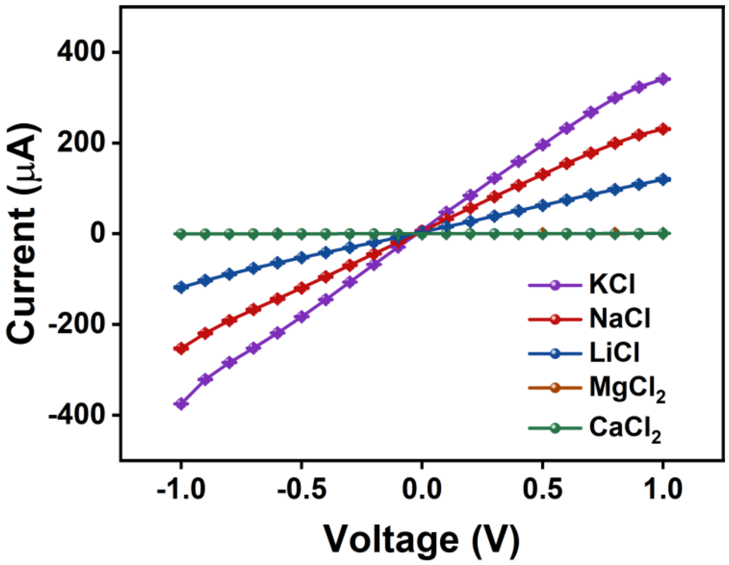

Supplementary Fig. 27 I-V curves of a UiO-66-$(OMe)_2$ membrane in different 100 mM electrolyte solutions as indicated. The error bars in the figure represent the standard deviation of three parallel tests.

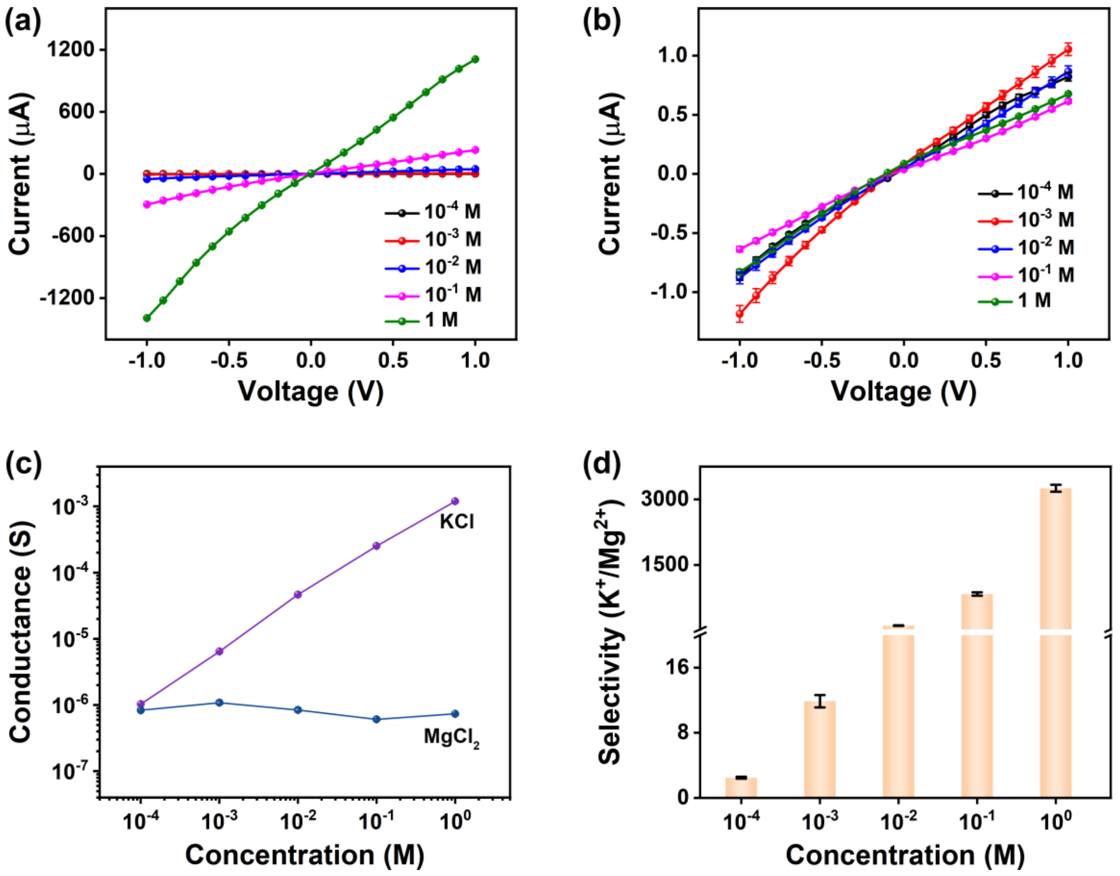

Supplementary Fig. 28 Relationship between ion selectivity and ion concentration in UiO-66-(OMe)₂ membranes. (a-b) I-V curves of a UiO-66-(OMe)$_2$ membrane in KCl and $MgCl_2$ solutions with various concentrations (10^{-4}-1 M). (c) Ionic conductance of the UiO-66-(OMe)$_2$ membrane in KCl and $MgCl_2$ solutions with various concentrations. (d) K^+/Mg^{2+} ion selectivity of a UiO-66-(OMe)$_2$ membrane at different concentrations (10^{-4}-1 M). The error bars in all figures represent the standard deviation of three parallel tests.

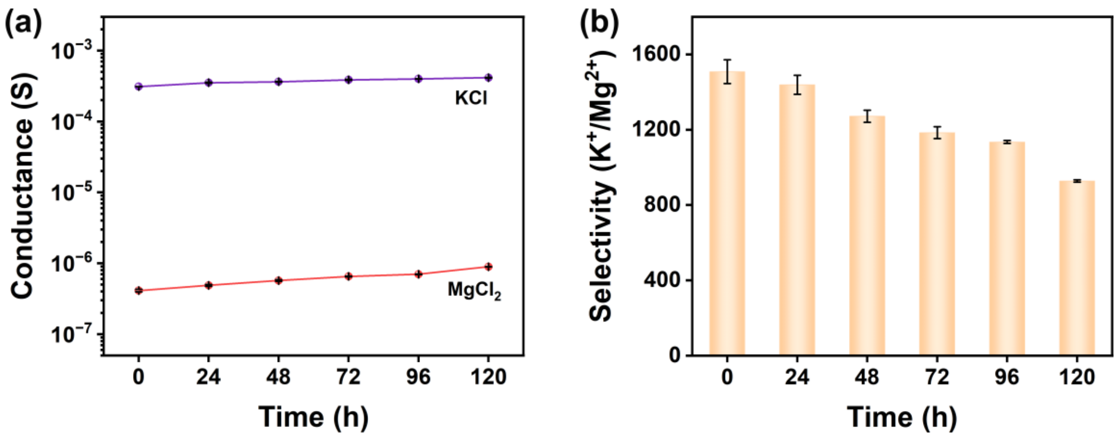

Supplementary Fig. 29 Stability of ion separation performance of UiO-66-$(OMe)_2$ membrane. (a) Relationship between conductance and time. (b) Relationship between ion selectivity and time. The error bars in all figures represent the standard deviation of three parallel tests.

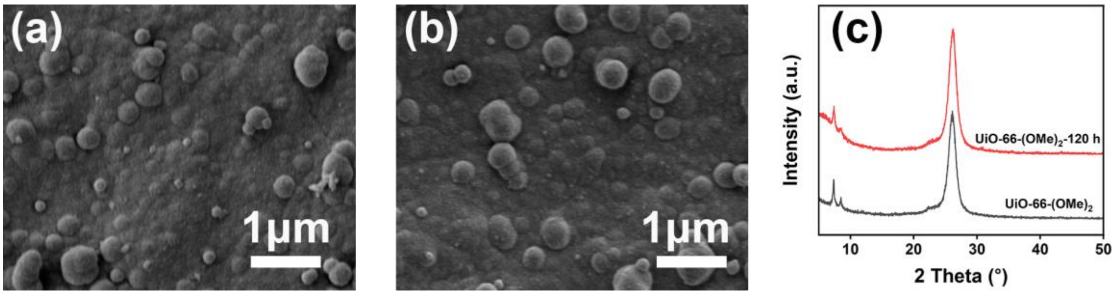

Supplementary Fig. 30 SEM images and XRD patterns of UiO-66-(OMe)$_2$ membrane after the stability test. (a) Initial surface morphology of the membrane. (b) Surface morphology of the membrane after 120 h stability test. (c) XRD patterns of the membrane before and after stability test.

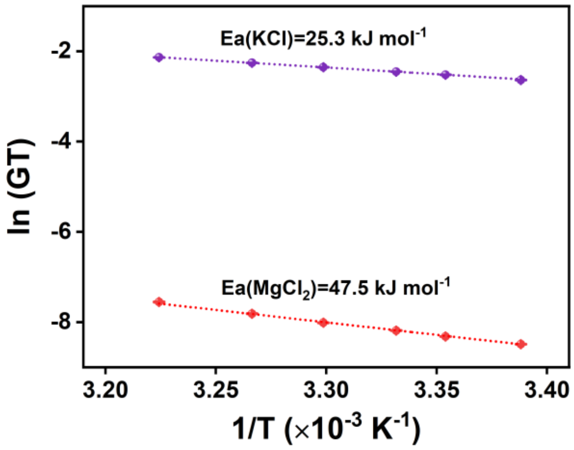

Supplementary Fig. 31 Arrhenius plots for a UiO-66-$(OMe)_2$ membrane in 100 mM KCl and $MgCl_2$ solutions. The error bars in this figure represent the standard deviation of three parallel tests.

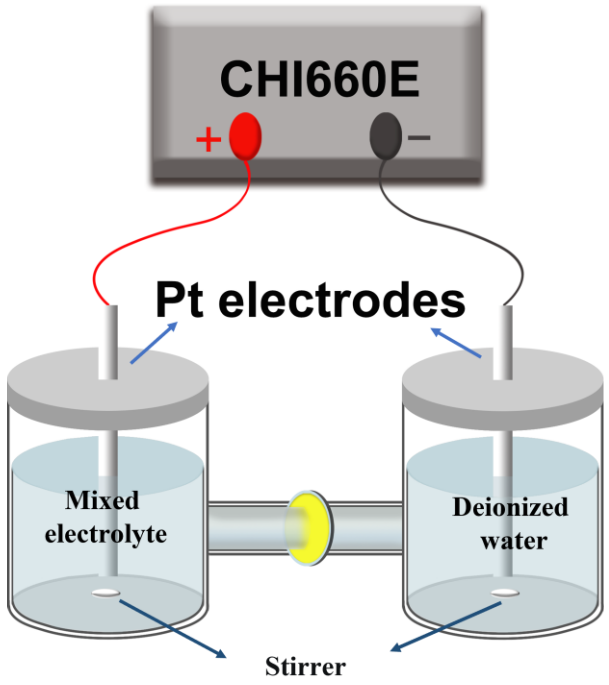

Supplementary Fig. 32 Schematic illustration of the device used ion permeation experiment.

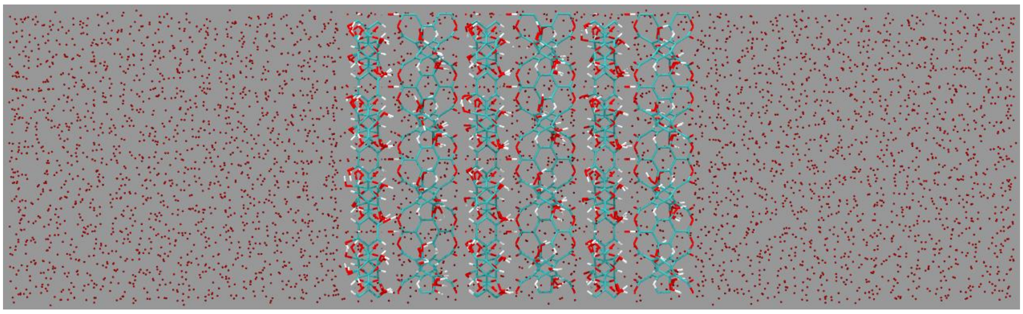

Supplementary Fig. 33 Model of UiO-66-$(OH)_2$ membrane used in MD simulation.

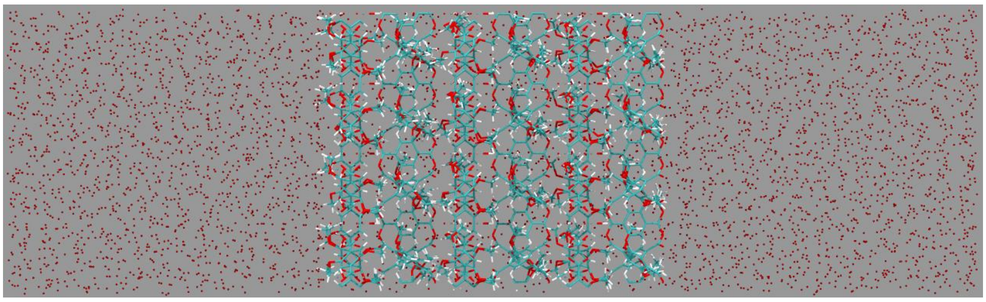

Supplementary Fig. 34 Model of UiO-66-$(OMe)_2$ membrane used in MD simulation.

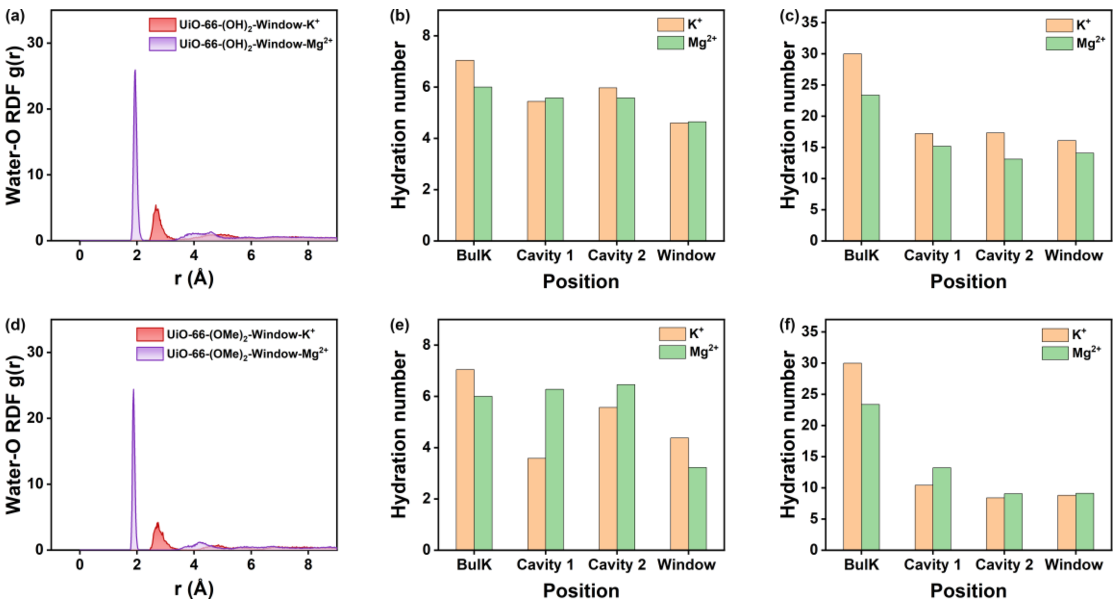

Supplementary Fig. 35 Radial distribution function (RDF) calculations of oxygen in water molecules. (a) RDF profiles of ion-water for K^+ and Mg^{2+} ions at the window region of UiO-66-$(OH)_2$. The first (b) and second (c) hydration shell hydration numbers of K^+ and Mg^{2+}ions in bulk, “Cavity 1”, “Cavity 2”, and “Window” regions of UiO-66-$(OH)_2$ membrane. (d) RDF profiles of ion-water for K^+ and Mg^{2+} ions at the window region of UiO-66-$(OMe)_2$. The first (e) and second (f) hydration shell hydration numbers of K^+ and Mg^{2+} ions in bulk, “Cavity 1”, “Cavity 2”, and “Window” regions of UiO-66-$(OMe)_2$ membrane.

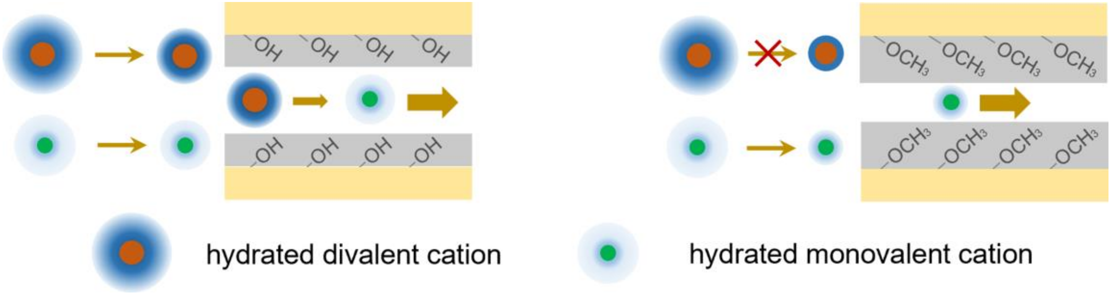

Supplementary Fig. 36 Schematic illustration of ion transport through UiO-66-$(OH)_2$ and UiO-66-$(OMe)_2$ membranes.

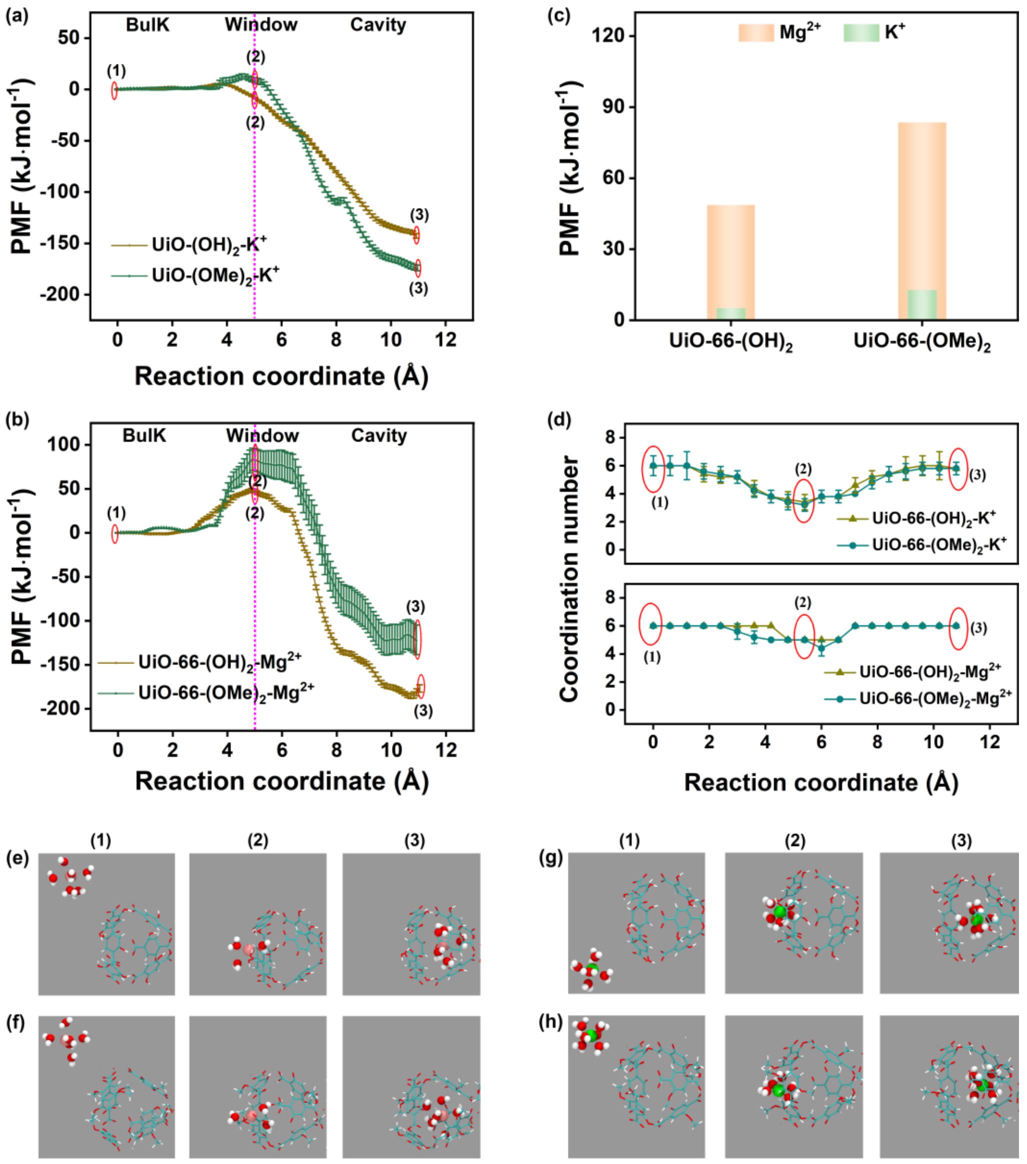

Supplementary Fig. 37 Energy barrier and dehydration effect of K^+ and Mg^{2+} in UiO-66-$(X)_2$. (a) PMF of metal ions migrating from solution through UiO-66-$(OH)_2$ frame; (b) PMF of metal ions migrating from solution through UiO-66-$(OMe)_2$ framework; (c) Energy barrier required for ions to pass through UiO-66-$(OH)_2$ and UiO-66-$(OMe)_2$ frames, respectively; (d) Coordination number of K^+ and Mg^{2+} entering UiO-66-$(OH)_2$ and UiO-66-$(OMe)_2$ frames from solution. The hydration state of K^+(e) and Mg^{2+}(g) ions in solution, UiO-66-$(OH)_2$ window and UiO-66-$(OH)_2$ cavity; Ionic hydration state of K^+(f) and Mg^{2+}(h) ions in solution, UiO-66-$(OMe)_2$ window and UiO-66-$(OMe)_2$ cavity. The error bars in (a) and (b) represent the standard deviation of the data collected in the final 6 ns of the simulation performed at each location. The error bars in (d) represent the standard deviation of five parallel tests.

2. Supplementary Tables

Supplementary Table 1. Binding energies between MOFs and ions calculated by DFT.

Binding energy / MOF	K^+ (eV)	Na^+ (eV)	Li^+ (eV)	Mg^{2+} (eV)	Ca^{2+} (eV)
UiO-66-$(NH_2)_2$	-0.529			-2.631	
UiO-66-$(NH_3^+Cl^-)_2$*	0.196			0.448	
UiO-66-$(SH)_2$	-0.493			-2.410	
UiO-66-$(OH)_2$	-0.668	-0.890	-1.183	-2.958	-1.394
UiO-66-$(OMe)_2$	-0.361			-1.843	

* The binding energies between UiO-66-$(NH_3^+Cl^-)_2$ and ions were calculated because UiO-66-$(NH_2)_2$ is positively charged in electrolyte solutions.

Supplementary Table 2. Properties of metal ions.

ion	Hydrated diameter (Å) [1]	Bare diameter (Å) [1]	Hydration free energy (kJ mol^{-1}) [2]
K^{+}	6.62	2.66	-295
Na^{+}	7.16	1.90	-365
Li^{+}	7.64	1.20	-475
Mg^{2+}	8.56	1.30	-1830
Ca^{2+}	8.24	1.98	-1505

Supplementary Table 3. Performance comparison of ion separation membranes.

Membrane	Transport rate (K^+) (mol m^{-2} h^{-1})	Ion selectivity (K^+/Mg^{2+}, Na^+/Mg^{2+}, Li^+/Mg^{2+})	Test system	Ref.
*Asy-MOFSNC	5.3×10^5	822.7, 336.7, 197.6	Binary cations	3
$TpBDMe_2$ membranes	0.207	213.4, 96.2, 35.8	Binary cations	4
*UiO-67	20.0	95.2, 61.9, 159.4	Unary cation	2
*PET Hosaphan film	0.00226,	39.6， 60.6, 634.0	Unary cation	5
Physically confined GO membrane	0.0065	650, 420, 500	Unary cation	6
Mxene	0.94	5.88, 9.56, 8.75	Unary cation	7
PIM-BzMA-TB	1.22	31.3, 32.6, /	Unary cation	8
DMBP-TB	0.20	48.8, 14.6, /	Unary cation	8
PIM@ZIF-8	0.031	9.69,14.84, 196.88	Five cations	9
FGOM-60	0.002	90,40, /	Unary cation	10
*CC3	0.87	163, 122, 104	Binary cations	11
GO-PEI	0.474	33.8, 27.0, 21.9	Unary cation	12

*PSS@HKUST-1	0.10	27.1, 51.9, 1815	Binary cations	13
*PET Lumirror film	0.034	16.7, 20.8, 21.1	Binary cations	14
Nitrogen-doped graphene membranes	0.1	390, 80, /	Unary cation	15
rGO membrane	0.19	169, 48, 12	Binary cations	16
*UiO-66-SO_3H	1.08	30, 138, 19	Binary cations	17
*UiO-66-NH_2	/	-, 210, 65	Binary cations	18
GO-PPD	0.09	7.15，4.8, /	quaternary ions	19
*UiO-66-$(OMe)_2$	0.05	1048.4, 707.3, 202.8	Five cations	This work

* represents that the driving force is electric field.

3. Supplementary references

1. Nightingale, E. R., Jr. Phenomenological theory of ion solvation. Effective radii of hydrated ions. *J. Phys. Chem.* **63**, 1381-1387 (1959).

2. Xu, R.M., Kang, Y., Zhang, W.M., Zhang, X.W. & Pan, B.C. Oriented UiO-67 metal-organic framework membrane with fast and selective lithium-ion transport. *Angew. Chem.-Int. Edit.* **61**, e202115443 (2022).

3. Lu, J. et al. Efficient metal ion sieving in rectifying subnanochannels enabled by metal-organic frameworks. *Nat. Mater.* **19**, 767-774 (2020).

4. Sheng, F.M. et al. Efficient ion sieving in covalent organic framework membranes with sub-2-nanometer channels. *Adv. Mater.* **33**, 2104404 (2021).

5. Wen, Q. et al. Highly selective ionic transport through subnanometer pores in polymer films. *Adv. Funct. Mater.* **26**, 5796-5803 (2016).

6. Abraham, J. et al. Tunable sieving of ions using graphene oxide membranes. *Nat. Nanotechnol.* **12**, 546-550 (2017).

7. Ren, C.E. et al. Charge- and size-selective ion sieving through Ti_3C_2Tx MXene membranes. *J. Phys. Chem. Lett.* **6**, 4026-4031(2015).

8. Tan, R. et al. Hydrophilic microporous membranes for selective ion separation and flow-battery energy storage. *Nat. Mater.* **19**, 195-202 (2020).

9. Kazemzadeh, H., Karimi-Sabet, J., Darian, J.T. & Adhami, A. Evaluation of polymer inclusion membrane efficiency in selective separation of lithium ion from aqueous solution. *Sep. Purif. Technol.* **251**, 10 (2020).

10. Qian, Y.J. et al. Enhanced ion sieving of graphene oxide membranes via surface amine functionalization. *J. Am. Chem. Soc.* **143**, 5080-5090 (2021).

11. Xu, T.T. et al. Highly ion-permselective porous organic cage membranes with hierarchical channels. *J. Am. Chem. Soc.* **144**, 10220-10229 (2022).

12. Huang, Q.B., Liu, S., Guo, Y.A., Liu, G.P. & Jin, W.Q. Separation of mono-/di-valent ions via charged interlayer channels of graphene oxide membranes. *J. Membr. Sci.* **645**, 120212 (2022).

13. Guo, Y., Ying, Y.L., Mao, Y.Y., Peng, X.S. & Chen, B.L. Polystyrene sulfonate threaded

through a metal-organic framework membrane for fast and selective lithium-ion separation. *Angew. Chem.-Int. Edit.* **55**, 15120-15124 (2016).

14. Wang, P.F. et al. Ultrafast ion sieving using nanoporous polymeric membranes. *Nat. Commun.* **9**, 569 (2018).

15. Song, J.H., Yu, H.W., Ham, M.H. & Kim, I.S. Tunable ion sieving of graphene membranes through the control of nitrogen-bonding configuration. *Nano Lett.* **18**, 5506-5513 (2018).

16. Xi, Y.H. et al. Graphene-based membranes with uniform 2D nanochannels for precise sieving of mono-/multi-valent metal ions. *J. Membr. Sci.* **550**, 208-218 (2018).

17. Xu, T.L. et al. Engineering leaf-like UiO-66-SO_3H membranes for selective transport of cations. *Nano-Micro Lett.* **12**, 11 (2020).

18. Xu, T.T. et al. Highly cation permselective metal-organic framework membranes with leaf-like morphology. *ChemSusChem* **12**, 2593-2597 (2019).

19. Jia, Z. Q.; Wang, Y.; Shi, W. X. & Wang, J. L. Diamines cross-linked graphene oxide free-standing membranes for ion dialysis separation. *J. Membr. Sci.* **520**, 139-144 (2016)
